# Tomek Link and SMOTE Approaches for Machine Fault Classification with an Imbalanced Dataset

**DOI:** 10.3390/s22093246

**Published:** 2022-04-23

**Authors:** Elsie Fezeka Swana, Wesley Doorsamy, Pitshou Bokoro

**Affiliations:** 1 Department of Electrical and Electronics Engineering Technology, Doornfontein Campus, University of Johannesburg, Johannesburg 2028, South Africa; eswana@uj.ac.za (E.F.S.); pitshoub@uj.ac.za (P.B.); 2 Institute for Intelligent Systems, Auckland Park Campus, University of Johannesburg, Johannesburg 2006, South Africa

**Keywords:** imbalanced data, Bayesian classification, support vector machine, k-nearest neighbor, Tomek link, synthetic minority over-sampling sampling, wound-rotor induction generator

## Abstract

Data-driven methods have prominently featured in the progressive research and development of modern condition monitoring systems for electrical machines. These methods have the advantage of simplicity when it comes to the implementation of effective fault detection and diagnostic systems. Despite their many advantages, the practical implementation of data-driven approaches still faces challenges such as data imbalance. The lack of sufficient and reliable labeled fault data from machines in the field often poses a challenge in developing accurate supervised learning-based condition monitoring systems. This research investigates the use of a Naïve Bayes classifier, support vector machine, and k-nearest neighbors together with synthetic minority oversampling technique, Tomek link, and the combination of these two resampling techniques for fault classification with simulation and experimental imbalanced data. A comparative analysis of these techniques is conducted for different imbalanced data cases to determine the suitability thereof for condition monitoring on a wound-rotor induction generator. The precision, recall, and f1-score matrices are applied for performance evaluation. The results indicate that the technique combining the synthetic minority oversampling technique with the Tomek link provides the best performance across all tested classifiers. The k-nearest neighbors, together with this combination resampling technique yielded the most accurate classification results. This research is of interest to researchers and practitioners working in the area of condition monitoring in electrical machines, and the findings and presented approach of the comparative analysis will assist with the selection of the most suitable technique for handling imbalanced fault data. This is especially important in the practice of condition monitoring on electrical rotating machines, where fault data are very limited.

## 1. Introduction

Rotating electrical machines are essential equipment in industries such as wind turbines, compressors, gearboxes, cranes, motors, generators, power plants, etc., across several different applications [1]. Regardless of design improvements, operation, and maintenance of rotating electrical machines over the years, in practice, these machines are still vulnerable to a variety of faults which may lead to production and revenue losses due to unscheduled maintenance and repairs [2]. Condition monitoring, in the form of predictive maintenance, is a desirable capability that enables online and incipient fault detection [3]. The most common problems occurring in induction machines are inter-turn faults on stator and rotor windings, broken rotor bars and end rings, static and dynamic air-gap irregularities, bowed shaft, bearings misalignment, and mechanical imbalances [4]. Modern condition monitoring methods may be broadly categorized into model-based and data-driven approaches. Although model-based methods have been successfully applied in practice over the years, new techniques continue to be proposed to further improve and progress the field. Model-based methods are based on physics and mathematical modeling to outline the machine’s fault type and prior assumptions of various physics parameters are required. These techniques are typically based on parameter estimations, parity equations, and state observers [5]. The model-based techniques generally operate by using a threshold on generated residual signals to detect faults. Once the threshold is exceeded, the fault can be isolated. This approach has the advantage that it can provide high accuracy, easy interpretation, and clear analysis, and does not require large amounts of historical data. However, they have limitations, namely—assumptions about the system need to be made, prior knowledge of the exact physical processes and failure mechanism is required to build an expert system, and accuracy and robustness inherently depend on the model development conditions [6]. On the other hand, data-driven methods based on machine learning and the feature extraction process could be either statistical or non-statistical, and they require data generated under various conditions. Despite their numerous advantages, data-driven methods, particularly supervised-learning fault classifiers, are not used widely in practice due to the problem of a lack of adequate fault-condition data as compared to healthy condition data.

This paper aims at overcoming the challenges that data imbalances poss to supervised-learning-based condition monitoring on a WRIG. This research specifically deals with classification stator and rotor winding inter-turn short-circuits and brush faults on a WRIG. The supervised-learning classifiers, namely, Naïve Bayes Classifier (NBC), Support Vector Machines (SVM), and k-nearest neighbors (k-NN), together with synthetic minority over-sampling technique (SMOTE) and Tomek link (T-link) methods, are applied on combined features extracted from multiple electrical signals—i.e., stator voltage and current and rotor current signals. A comparative analysis of the aforementioned approaches is presented when dealing with various levels of imbalanced data. The performances are then comprehensively evaluated through several key measures, namely, precision, recall, and F-measure. The presented research is intended to address progress in the development of data-driven approaches for the condition monitoring of generators.

In this investigation, SMOTE and T-link will be implemented on multiple simulated and experimental data of the WRIG. To the best of the authors’ knowledge, an investigation into addressing fault data imbalances for data-driven condition monitoring on a WRIG has not yet been presented and will certainly contribute to condition monitoring practice and the growing knowledge in this area.

The rest of the paper is organized as follows. A brief motivation for the presented study is presented. Thereafter, a review of the investigated resampling approaches—that is, the Tomek link (T-link) and Synthetic minority over-sampling technique (SMOTE) are briefly presented. The methodology including the machine modeling, feature extraction, and application of the techniques are then presented before a detailed comparison, integrated techniques, and interpretation of the results is given. Finally, a summary of the key findings of the research is presented in Section 5.

## 2. Background

### 2.1. Overview

Data-driven methods are primarily based on machine learning, which necessitates data generated under various conditions to be diagnosed. The generated data enable an automatic fault detection and diagnosis to be constructed [7]. For instance, the literature shows that the application of deep learning neural networks reduces manual labor and expect knowledge [8,9]. The data-driven approaches are based on historical data and can be classified into supervised, unsupervised, self-supervised, semi-supervised, and reinforcement learning condition monitoring. While supervised-learning condition monitoring methods are based on training and classifying with labeled data to predict unlabeled data [10], unsupervised learning methods can extract information and apply hidden patterns based on input data to produce a model from unlabelled data. Self-supervised learning is a relatively new approach that learns representative examples, which automates the supervisory signals from unlabelled input datasets and predicts the remaining input dataset. The self-supervised capability to learn unlabelled data allows it to perform big data analysis, which makes it attractive for condition monitoring [11] but requires further research development for practicable application. When it comes to rotating electrical machines, specifically generators—large amounts of condition data are generated, which can be employed by data-driven approaches for predictive maintenance purposes. Data-driven methods are becoming more attractive due to their flexibility, ease of development, and relatively lower costs. Additionally, these approaches are also well suited under real-time constraints [8].

The commonly used supervised-learning methods include artificial neural networks (ANN), support vector machine (SVM), Naïve Bayes classifier (NBC), and decision tree [11]. The application of these supervised-learning methods have been applied successfully and presented to be effective for condition monitoring in electrical machines. However, the effectiveness of the above-mentioned methods has been based on the assumption that each class has been presented with the same number of instances. An experimental setup may be built to generate balanced data for various machine conditions. However, in practice, the machine mostly operates under healthy conditions and that results in abundantly healthy data being collected. The faulty data will only be generated when the machine experiences some faults, and there are significantly lower numbers of faulty data instances compared to healthy data. This results in the different numbers of instances or observations for the various classes, and this is referred to as an imbalanced dataset. This imbalanced dataset may lead to misclassification. Various methods have been proposed to reduce the data imbalance challenges, namely, the resampling (under-sampling of majority instances/over-sampling of minority instances) algorithm technique and ensemble methods, together with algorithm approaches for the enhancement of classifiers [12].

Chawla proposed a synthetic minority over-sampling technique (SMOTE) which is based on the k-nearest neighbor to generate new instances [13]. Sun [14] proposed an integrated method that includes SMOTE with AdaBoost support vector machine with time weighting for financial distress data. In [15], the data sampling method together with logistic regression, SVM, and k-NN were applied to an imbalanced cardiac surgery dataset. The results presented were based on the original data, undersampling, and oversampling, with very poor sensitivity of the logistic regression, SVM, and kNN based on the original data. With the application of the sampling methods, the results improved.

The cluster MWMOTE was proposed to overcome the limitations of oversampling techniques (SMOTE, ADASYN) based on k-NN, which are overgeneralization, noise, sensitivity, and missing some boundary instances [16]. However, MWMOTE does not improve the boundary instances [17]. In addition, the minority class separation is ignored. In [18], the sample-characteristic oversampling technique (SCOTE) based on LS-SVM was proposed for bearing faults diagnosis with an imbalanced dataset. The SCOTE filters out the noisy points by applying k-NN-based noise processing and then trained with LS-SVM. In [19], the comparison of naïve Bayes classifier (NBC), decision tree, and Adaboost algorithm together with SMOTE techniques was implemented for rotor fault on an induction motor. The AdaBoost method was presented to be performing better compared to the other two methods, and NBC has been presented to have the worst performance. However, as the severity of the fault increased, AdaBoost showed poor performance results as presented by performance metrics. In addition, after the SMOTE application, the performance of each classifier improved. The ROC curve performance evaluation presented that AdaBoost outperforms the NBC and decision tree. However, the application of these aforementioned methods to handle the imbalanced dataset is very limited when it comes to condition monitoring in electrical machines, specifically the wound-rotor induction generators (WRIG).

### 2.2. Imbalanced Dataset

When the imbalanced datasets are presented, one class has majority instances and the other classes have minority instances. This results in an uneven distribution of classes and misclassification of minority instances as the classifier system tends to be biased and in favor of the majority instances [20]. The classifier system also tends to ignore the minority classes and detect them as noise [21]. When it comes to electrical machines, the majority of instances are associated with the healthy class and the minority instances are associated with various faults.

The data imbalance strategies such as resampling, cost-sensitive learning, and ensemble were developed to work together with various data-driven techniques to handle the imbalanced data. The recall, precision, F-measure, G-mean, and receiver operating characteristics (ROC) curves are the commonly used assessment performance metrics for data imbalance [12].

### 2.3. Intelligent Approaches

Supervised learning depends on trained and labeled data to predict unlabelled data. The supervised-learning technique is the most commonly used machine learning in electrical machines for the improvement of data imbalance challenges.

Bayesian classification is a supervised learning technique that applies logical calculus for making decisions under uncertainty. The key benefit of Bayesian classification is its strong theoretical foundation and mathematical computation to make predictions. Bayesian classification employs Bayes’ theorem, which is an algebraic model based on the fundamentals of probability theory [22]. The additional benefit of NBC includes its time efficiency, CPU usage, and memory. NBC also applies strong independence assumptions and works using an independent feature model [23].

A support vector machine (SVM) is a classifier that aims at determining the hyperplane in linear classification. SVM works well in handling a small amount of data and can improve accuracy. In this method, every single data point is presented as a vector and with each of these data points belonging to two different classes the maximum distance between these points contributes to accuracy and in determining the best hyperplane. For nonlinear classification, SVM employs a kernel machine that replaces the data points. The kernel machine does not require prior information and its computation is simple. However, kernel machine takes longer to process large datasets [11].

### 2.4. Resampling Techniques

Resampling aims at equalizing the number of instances per class either reducing the majority class instances, known as under-sampling, or increasing the minority class instances, known as over-sampling. Under-sampling techniques reduce the majority instances by randomly eliminating majority class instances [24]. With the advantage that it can improve run time and storage problems. However, it can eliminate important data. The remaining data may be a biased sample and unable to provide accuracy for classes distribution [20,24]. Various under-sampling methods have been proposed, and the most commonly used are random under-sampling and Tomek link (T-link).

Tomek developed the Tomek link, which was originally designed for two different classes (one majority and one minority), where, if the majority and minority classes are $x_{a}$ and $x_{b}$, then the distance between them will be $d\left(x_{a},x_{b}\right)$ and is known as the Tomek link, provided that no other class $x_{z}$ such that $d\left(x_{a},\;x_{z}\right)<d\left(x_{a},x_{b}\right)$ or $d\left(x_{b},\;x_{z}\right)<d\left(x_{a},x_{b}\right)$ [25]. T-link works by eliminating the majority class instances that are closer to the minority class by applying the nearest neighbor rule to select instances [26]. T-link is also classified as an improved condensed nearest neighbor [27]. This method can also be applied for post-processing cleaning data when instances from the majority and minority classes are removed, which is due to the lack of well-defined borderline regions. This method can be an under-sampling only when the majority class instances are removed [25]. T-link was applied together with a random forest classifier for the prediction of depression symptoms based on their severity. The results presented to be very poor for some classes based on the evaluation matrices with original imbalanced data. After the T-link application, the results improved drastically. The T-link method was performed as post-process cleaning data. The hybrid of T-link and random oversampling presented improved accuracy compared to individual performances.

Over-sampling increases the minority instances by randomly replicating the minority class instances to a required level to represent a balanced class distribution [20,24]. This method has the advantage that no data are eliminated and it performs better compared to under-sampling. However, this method may lead to overfitting due to replicated instances. The synthetic minority over-sampling technique (SMOTE) is an improved oversampling technique that was developed by Chawla [13]. SMOTE is based on a k-nearest neighbor to generate new synthetic sampling in feature space based on a certain percentage for the minority classes. SMOTE can generate new synthetic data based on the existing minority class data without replicating it to overcome the overfitting challenge. This synthetically generated data can be formulated as given in Equation (1):

(1) $$S_{syn}=r\left(S_{kNN}-S_{f}\right)+S_{f}.$$

where *S_syn_*—generated synthetic samples; *S_f_*—feature samples; *S_kNN_*—considered feature sample k-nearest neighbor; and *r*—a random number between 0 and 1. The classifier develops specific regions based on the synthetic samples.

When it comes to rotating machines, the SMOTE has been limited to induction motor faulty rotor bars where NBC, decision tree, and Adaboost algorithm together with SMOTE techniques were compared. After the SMOTE application, the performance of each classifier improved [19]. In this paper, SMOTE and T-link are both used with multiple data generated from both the simulation and experimental work of WRIG. The generator is operated at a resistive load of 300 Ω per phase conditions.

### 2.5. Assessment Metrics

The assessment metrics are based on the confusion matrix, which presents the true positive and true negative classes as shown in Table 1. The metrics are well-defined by Equations (2)–(5) [12]:

**Table 1:** Confusion Matrix.

	Positive	Negative
**Positive**	True positive (*TP*)	False negative(*FN*)
**Negative**	False positive (*FP*)	True negative (*TN*)

(2) $$Recall=\frac{TP}{TP+FN}$$

(3) $$\mathrm{Precision}=\frac{TP}{TP+FP}$$

(4) $$F-measure=\frac{\left(1+\beta^{2}\right)\times Recall\times Precision}{\beta^{2}\times Recall+Precision}$$

(5) $$G-mean=\sqrt{\frac{TP}{TP+FN}\times \frac{TN}{TN+FP}}$$

where recall represents the correctly classified positive attributes and is not sensitive to data changes. In addition, the recall does not provide information with regards to the incorrectly positive labeled attributes [12]. Precision measures the actual correctly labeled attributes and is sensitive to data changes. Similar to recall, precision also does not provide information about incorrectly labeled instances. However, recall and precision have been presented to be effective with data imbalance. The F−measure is the combination of recall and precision as a measure of effectiveness in terms of the ratio of either recall or precision which is weighted by the coefficient β where β is the coefficient to vary the relative importance of precision against the recall. Although the F-measure is sensitive to data changes, it is capable to provide more information compared to accuracy and error rate metrics. The geometry mean (G-mean) metric evaluates the inductive bias degree in terms of correctly classified positive and negative attributes. G−mean performs better compared to the traditional metrics [20]. The receiver operating characteristics (ROC) curves employ single column-based evaluation metrics, which present the true positive rate and false-positive rate. The ROC can provide the threshold of true positives and false positives. The ROC also provides the function sensitivity values and all the points are joined together to form a graph [28]. The ROC’s performance is based on the inclined leaning towards the *y*-axis and the area under the curve analysis.

## 3. Methodology

### 3.1. Overview

Typically, the supervised learning classification in condition monitoring depends on the clear distribution of classes. In the case where the distribution of classes is imbalanced—the lack of availability of fault data, as compared to healthy operation data, conveys a challenge to detect which brings uncertainties to applying these methods. The presented methodology investigates the NBC, SVM, and k-NN performances based on simulated and experimental imbalanced data for condition monitoring of a WRIG using combined multiple signals. Then, the resampling methods, namely, SMOTE, T-link, and a combination of SMOTE and T-link are applied to the combined data. Whereas SMOTE is an oversampling method that replicates the minority classes, T-link is an under-sampling method, which, in this investigation, was performed as post-processing cleaning data. The classification techniques verified that with the application of resampling methods, the imbalanced data challenges based on condition monitoring can be reduced for a WRIG.

### 3.2. Process Description

**Figure 1:**
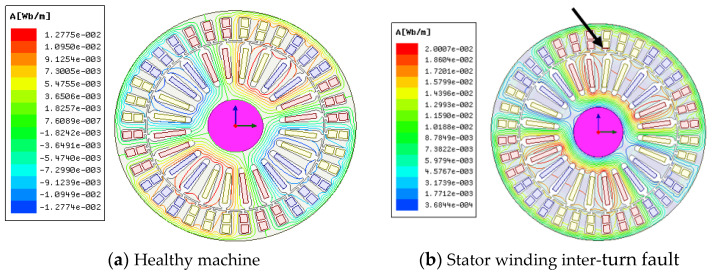

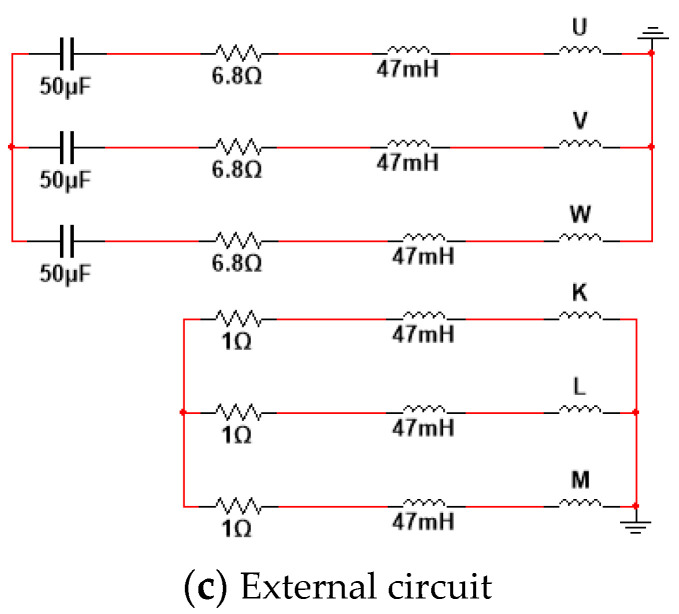
(**a**) Geometry of wound-rotor induction generator model with flux lines distribution; (**b**) Flux lines distribution with stator winding inter-turn fault indicated (by arrow); (**c**) External circuit of the investigated wound-rotor induction generator.

The 3-phase, 1 kW, 380 V, 4 pole wound-rotor induction machine model was created using ANSYS Maxwell. The geometry of the healthy model, indicating flux lines distribution amongst 4 poles, is presented in Figure 1a. Figure 1b presents the geometry with the stator inter-turn short circuit fault implemented in the simulations and the flux lines depict asymmetry distribution. The corresponding external circuit used for excitation of the WRIG is shown in Figure 1c.

The faults considered are inter-turn short circuits on the stator windings and the rotor windings, and brush faults. These faults are considered separately, and therefore, multiple models were created—i.e., healthy, stator fault, rotor fault, and brush fault. The WRIG machine has three-phase windings on both the rotor and stator. Faults are modeled through short-circuiting the turns of one of the phase coils. Three and six turns are short-circuited in each case to incorporate the different levels of the same fault type. The brush fault is simulated by connecting a 0.5 Ω resistor in series with the brush rotor in the external circuit. Different instances are obtained by randomly varying the external circuit excitation capacitor by ±2% [29].

**Figure 2:**
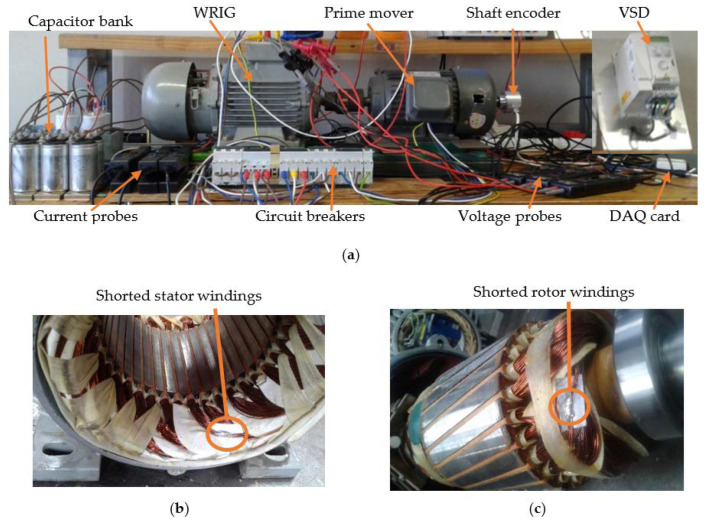
(**a**) Experimental layout; (**b**) Stator winding inter-turn fault; (**c**) Rotor winding inter-turn fault.

The WRIG experimental layout, the stator inter-turn short circuit, and rotor inter-turn faults modifications on the machine are presented in Figure 2a–c. The setup entails a three-phase, 1 kW, 380 V, 4 pole wound-rotor induction machine, capacitor bank, circuit breakers, prime mover, variable speed drive, variable resistors, voltage/current transducer, shaft encoder, and data acquisition card.

The WRIG model constructed is modified to account for the three considered fault conditions, which are the inter-turn short circuit in the stator windings, the inter-turn short circuit in the rotor windings, and the brush fault. The modification for inter-turn winding faults is implemented on the overhangs of the stator and the rotor. The 3.7 Ω variable resistor is connected in series with the brush for fault implementation.

### 3.3. Feature Extraction

**Figure 3:**
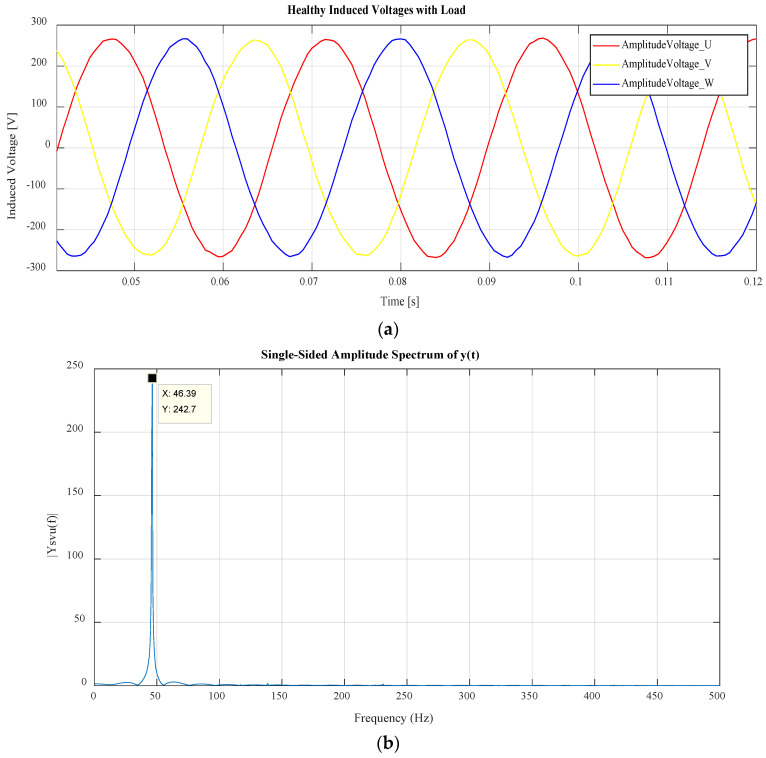
(**a**) Three-phase steady−state stator phase voltage shown for a portion of acquisition time used; (**b**) stator voltage phase U under healthy conditions.

The data attained through the simulation measurements are processed with the Fast-Fourier transform (FFT) applied to all the signals. In practice, these signals are simply recorded using voltage and current sensing. A sample of the stator voltage under healthy steady-state conditions for a portion of the acquisition time for the WRIG with load is presented in Figure 3a. Figure 3b presents the spectra with a two-second acquisition time for the measured stator voltage signals under healthy conditions, where the WRIG is operated at resistive load conditions of 300 Ω per phase at 1347 rpm speed.

These signals consist of 11 orders per phase including the DC component for both stator current and voltage, and 10 per phase for the rotor current. The 11 orders for each case start from and DC harmonic component and odd and even harmonics up to 500 Hz. Therefore, 33 various features were obtained for each data instance of stator phase voltage and current and 30 features for each instance of the rotor phase currents. The harmonics obtained from the FFT are then normalized with respect to the maximum and minimum harmonic for feature scaling, better harmonic resolution, and relative significance of the fundamental harmonic. Therefore, when normalized, the magnitude of all harmonics orders are calculated with respect to the fundamental. The fundamental harmonic component is equal to unity after normalization. Each harmonic for multiple signals is then extracted and used as features for various classification and resampling methods, namely, Bayesian classification, Support Vector Machines (SVM), and k-nearest neighbors (k-NN), together with Tomek link (T-link), synthetic minority over-sampling sampling (SMOTE), and combined SMOTE and T-link methods.

### 3.4. Classification Process

**Figure 4:**
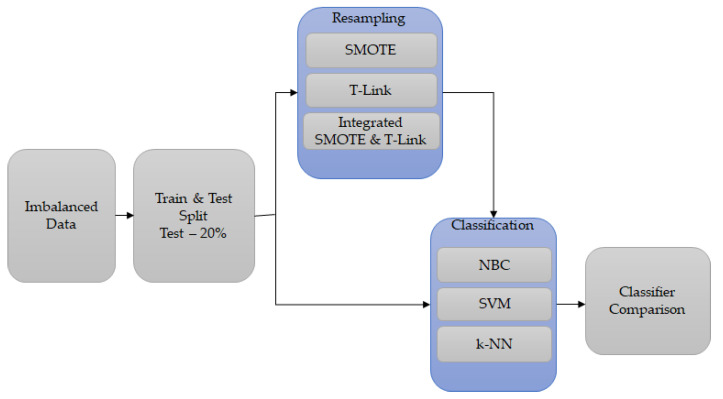
The method for WRIG imbalanced data.

The classification of the generator imbalanced data was performed with python anaconda for each scenario, that is, the stator voltage and current, and rotor current as presented in Table 2. These cases are for training purposes, and the faults may not occur at the same time. The investigated method is shown in Figure 4. The NBC, SVM, and k-NN classification methods were first applied to the original imbalanced simulated and experimental data cases. The data had a test split of 20% in each case. The resampling methods were applied individually which are SMOTE and T-link based on each data case. the integrated SMOTE and T-link method was also applied based on each case. The classification methods were reapplied to the resampled simulated and experimental data with the accuracy score, weighted averages of precision, recall, and F1-score results recorded.

## 4. Results and Analysis

### 4.1. Simulation Analysis

The NBC, SVM, and k-NN applications, together with SMOTE, T-link, and the combination of SMOTE/T-link for each case, are presented. The performance measures such as precision, recall, and F1-score are also presented. The weighted average of these performance measures ranges from 45% to 50% with the application of NBC on the original data for 20% of the test data. For various test data and cases, the improvement can be identified as presented in the figures below.

**Figure 5:**
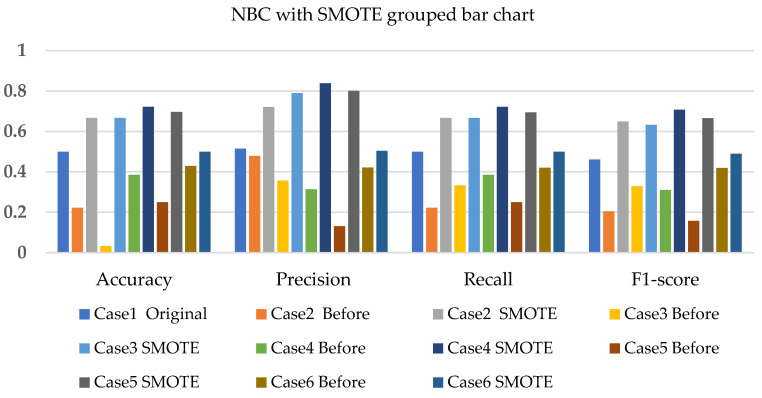
Simulation performance of NBC classification with SMOTE.

**Figure 6:**
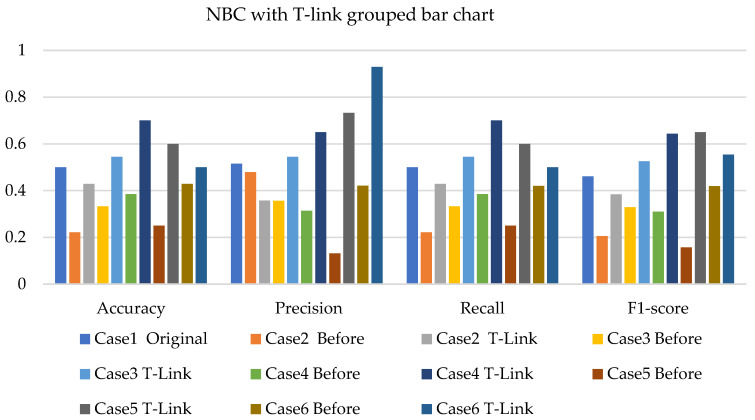
Simulation performance of NBC classification with T-link.

**Figure 7:**
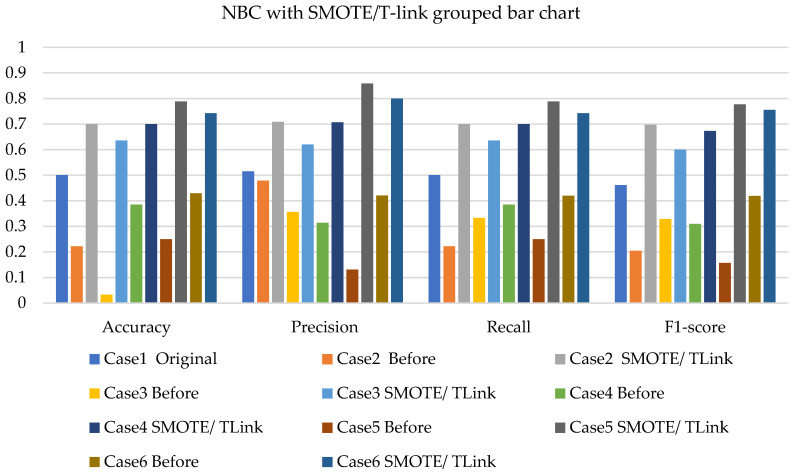
Simulation performance of NBC classification with SMOTE/T-link.

Figure 5, Figure 6 and Figure 7 present the NBC with SMOTE, NBC with T-link, and NBC with a combination of SMOTE/T-link. The NBC with SMOTE presents the highest accuracy and recall—0.722; precision—0.839; andF1-score—0.777 with data level case 4. With data level case 6 being the worst scenario with the accuracy and recall—0.5, precision—0.504; and F1-score—0.49. The NBC with SMOTE has a minimum of 50% and a maximum of 70%. The NBC with T-link on data level case 2 has the worst performance with an accuracy and recall of 42.9%, a precision of 35.7, and an F1-score of 38.4%. The data level case 4 has the best performance with 0.7—accuracy and recall; 0.65—precision; and 0.643—F1-score. Although the precision of case 6 is 0.929, its F1-score is 0.554, which presents a low performance. The NBC with T-link has a minimum accuracy of 40% and a maximum of 60%. The NBC with combined SMOTE/T-link presents improvements on all the data level cases of individual SMOTE. When combined compared to the individual performance of the T-link, most cases have drastically improved except case 3 with 0.636—accuracy and recall; 0.62—precision; and 0.6—F1-score performance below the individual T-link. The NBC with combined SMOTE/T-link has a minimum accuracy of 63% and a maximum of 78%.

**Figure 8:**
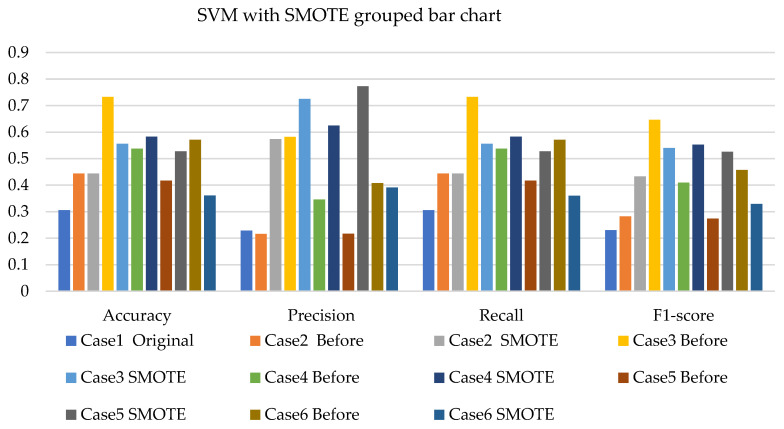
Simulation performance of SVM classification with SMOTE.

**Figure 9:**
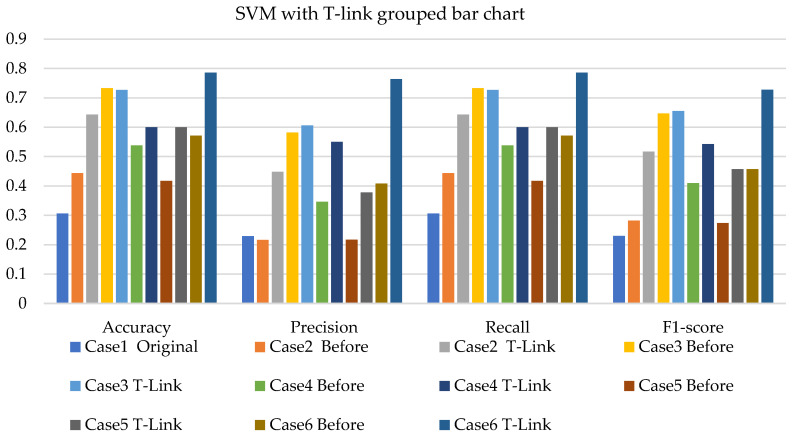
Simulation performance of SVM classification with T-link.

**Figure 10:**
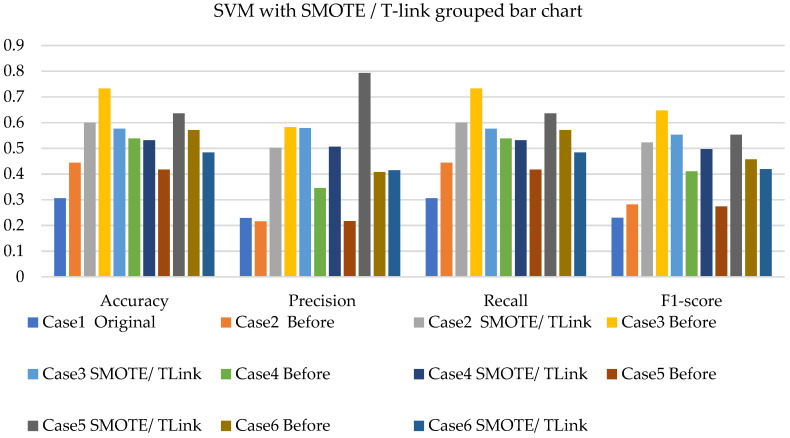
Simulation performance of SVM classification with SOMTE/T-link.

Figure 8, Figure 9 and Figure 10 present the SVM with SMOTE, SVM with T-link, and SVM with a combination of SMOTE/T-link. The SVM with SMOTE presents the best data level cases performance with slight improvements. Data level cases 3 and 6 show performances below the SVM without SMOTE with 17.7% and 21%—accuracy and recall; 14.3% and 21.1%—precision; and 10.79% and 12.8%—F1-score, respectively. The SVM with SMOTE has a minimum accuracy of 36% and a maximum of 58%. The SVM with T-link shows improvements in each case, with case 5 being the lowest, which performs with 0.6—accuracy and recall; 0.55—precision; and 0.543—F1-score. Case 6 shows the best performance with 0.786—accuracy and recall; 0.764—precision; and 0.728—F1-score. The SVM with T-link has a minimum accuracy of 60% and a maximum of 78%. In the SVM with combined SMOTE/T-link, most cases show slight improvements, with cases 3 and 6 present the poorest performances of 15.7% and 8.7%—accuracy and recall; 0.3% precision; and 12.4% and 3.8%—F1-score, respectively, which is below the classifier’s performance without the resampling technique. The SVM with combined SMOTE/T-link has a minimum accuracy of 48% and a maximum of 63%.

**Figure 11:**
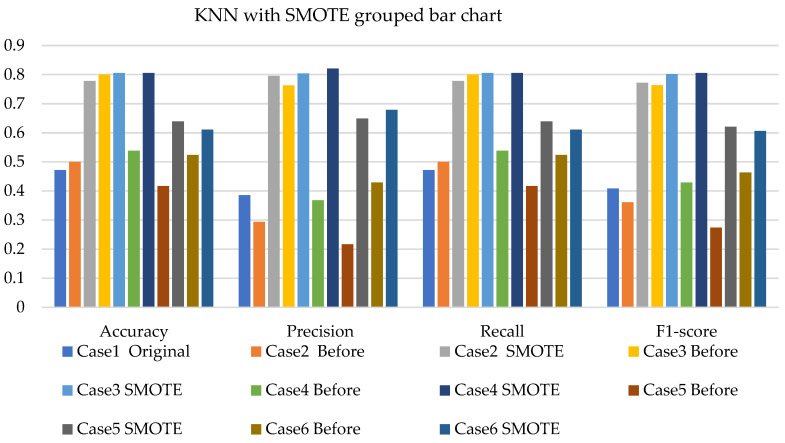
Simulation performance of k-NN classification with SMOTE.

**Figure 12:**
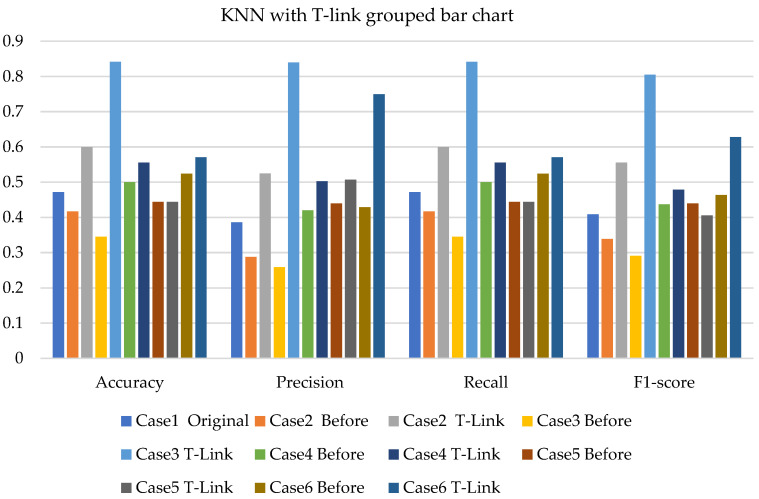
Simulation performance of k-NN classification with T-link.

**Figure 13:**
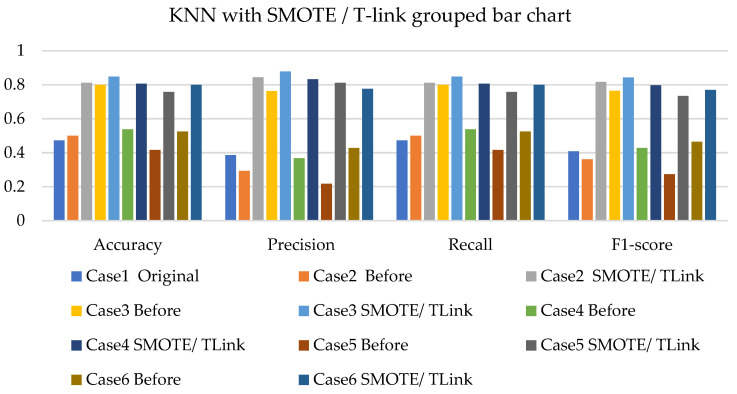
Simulation performance of k-NN classification with SMOTE/T-link.

Figure 11, Figure 12 and Figure 13 present the k-NN with SMOTE, k-NN with T-link, and k-NN with a combination of SMOTE/T-link. The k-NN with SMOTE presents improvements for all the cases, with case 4 showing better results of 0.806—accuracy and recall; 0.821—precision; and 0.806—F1-score. The k-NN with SMOTE has a minimum of 61% and a maximum of 80% accuracy. The k-NN with T-link presents improvements for most cases, excluding case 4, which presents a performance that is below the k-NN without T-link with 7.3%—accuracy and recall; 10.1%—precision; and 10.6%—F1-score. The k-NN with T-link has a minimum of 57% and a maximum of 70% accuracy. The k-NN with combined SMOTE/T-link shows improvements in all the cases, with case 3 showing the highest performance with 0.848—accuracy and recall; 0.878—precision; and 0.843—F1-score. Case 5 presents the lowest performance with 0.758—accuracy and recall; 0.811—precision; and 0.734—F1-score. Although case 5 has the lowest performance, the improvement is over 30% compared to k-NN without the SMOTE/T-link method. The k-NN with combined SMOTE/T-link has a minimum accuracy of 75% and a maximum of 84%.

### 4.2. Experimental Analysis

**Figure 14:**
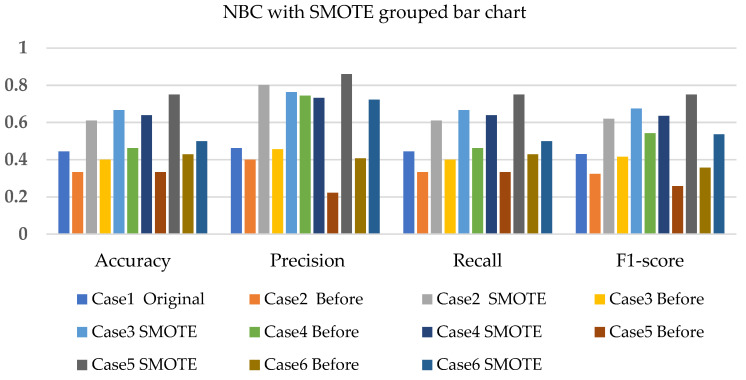
Experimental performance of NBC classification with SMOTE.

**Figure 15:**
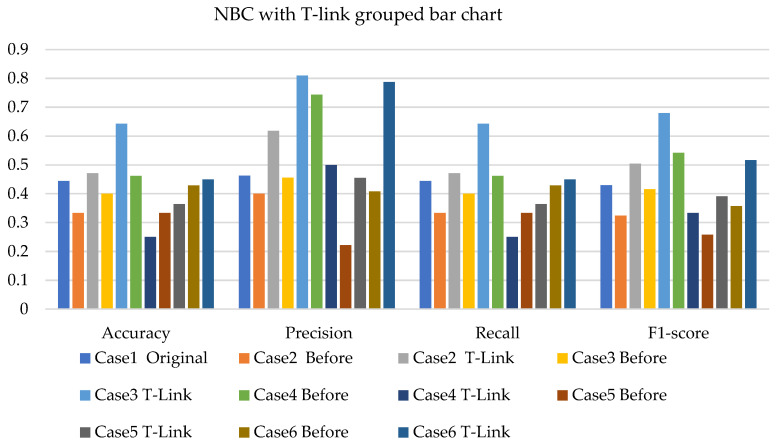
Experimental performance of NBC classification with T-link.

**Figure 16:**
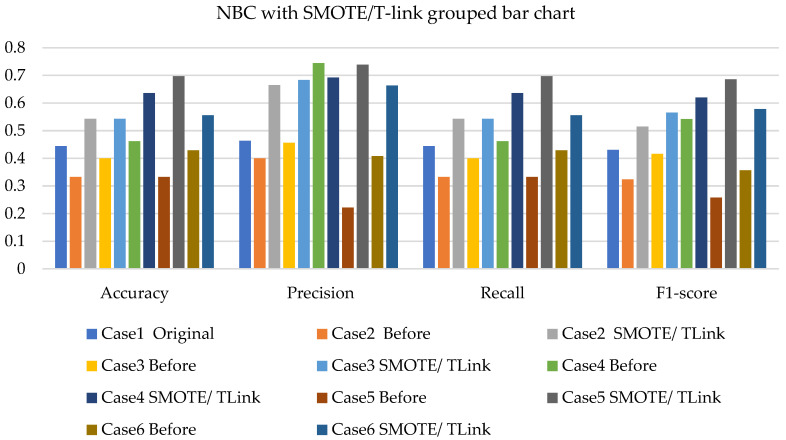
Experimental performance of NBC classification with SMOTE/T-link.

Based on the various cases with the split of 20% test experimental data. Figure 14, Figure 15 and Figure 16 present the weighted average performances of NBC with SMOTE, NBC with T-link, and NBC with SMOTE/T-link. The NBC with SMOTE presents case 5 with the best performance of 0.75—accuracy and recall; 0.86—precision; and 0.751—F1-score. The lowest performance is presented in case 6. The NBC with SMOTE has a minimum accuracy of 50% and a maximum of 75%. The NBC with T-link presents case 3 with the highest performance of 0.643—accuracy and recall; 0.81—precision; and 0.68—F1-score. Case 4 presents the poor performance of 0.25—accuracy and recall; 0.5—precision; and 0.333—F1-score, which is below the NBC without T-link. The NBC with T-link has a minimum accuracy of 25% and a maximum of 64%. The NBC with combined SMOTE/T-link presents case 5 with the highest performance of 0.697—accuracy and recall; 0.739—precision; and 0.686—F1-score. The NBC with combined SMOTE/T-link has a minimum accuracy of 51% and a maximum of 70%.

**Figure 17:**
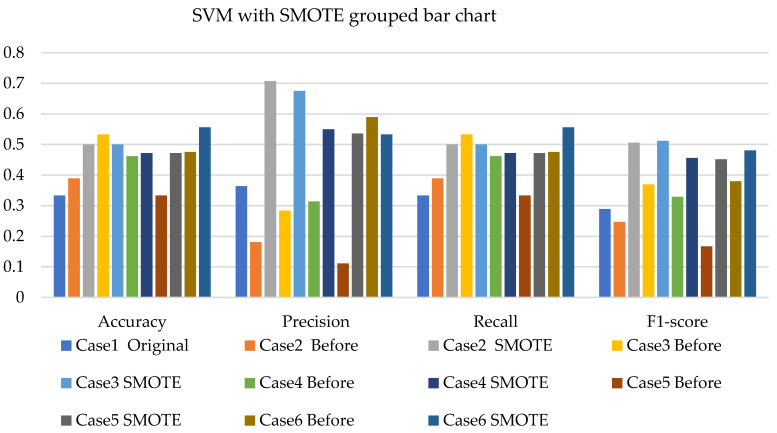
Experimental performance of SVM classification with SMOTE.

**Figure 18:**
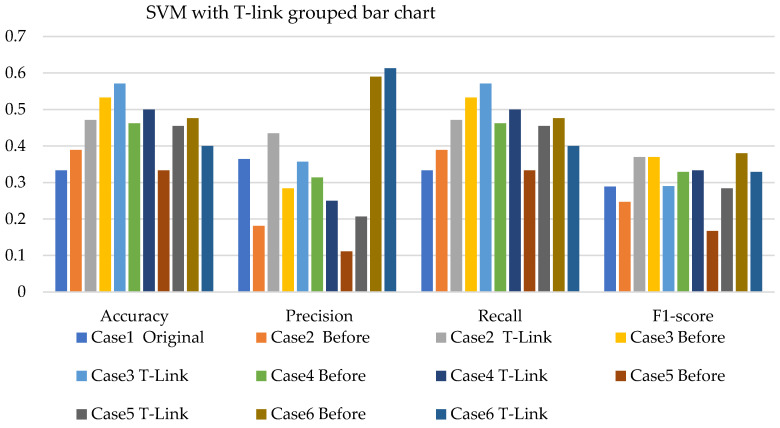
Experimental performance of SVM classification with T-link.

**Figure 19:**
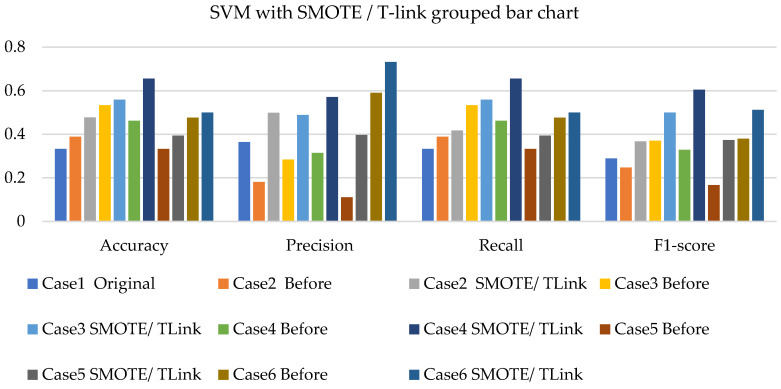
Experimental performance of SVM classification with SMOTE/T-link.

Figure 17, Figure 18 and Figure 19 present the weighted average performances of SVM with SMOTE, SVM with T-link, and SVM with SMOTE/T-link. The SVM with SMOTE presents case 2 having the highest performance of 0.5—accuracy and recall; 0.707—precision; and 0.506—F1-score. All other cases present slight improvements. The SVM with SMOTE has a minimum of 45% and a maximum of 56% accuracy. The SVM with T-link shows most of the cases with slight improvements, except case 6, which presents a performance with 16% lower accuracy and recall and a 13.4% lower F1-score than the performance of SVM without T-link. The SVM with T-link has a minimum accuracy of 40% and a maximum of 57%. The SVM with combined SMOTE/T-link presents each case with slight improvements. Case 4 has the highest performance with a 0.656 accuracy and recall, a 0.571 precision, and a 0.605 F1-score. Although case 6 precision is 0.732, which is higher, but the accuracy and F1-score are lower compared to case 4. The SVM with combined SMOTE/T-link has a minimum accuracy of 39% and a maximum of 65%.

**Figure 20:**
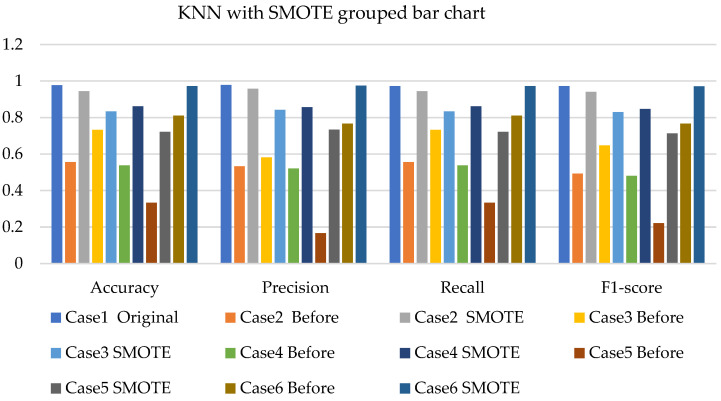
Experimental performance of k-NN classification with SMOTE.

**Figure 21:**
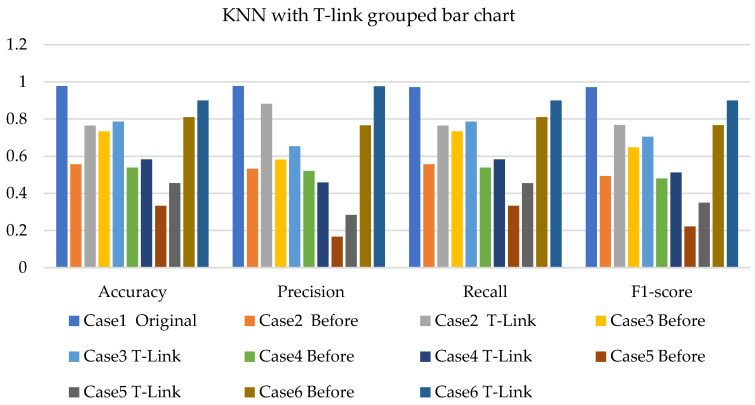
Experimental performance of k-NN classification with T-link.

**Figure 22:**
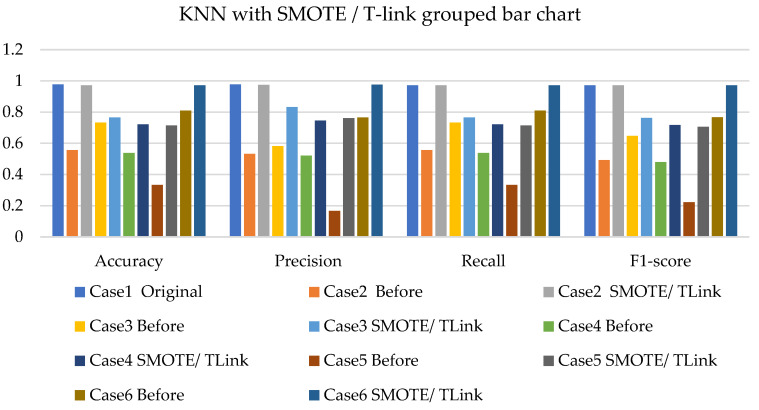
Experimental performance of k-NN classification with SMOTE/T-link.

Figure 20, Figure 21 and Figure 22 present the weighted average performances of k-NN with SMOTE, k-NN with T-link, and k-NN with SMOTE/T-link. The k-NN with SMOTE presents the highest performance of 0.972—accuracy and recall; 0.975—precision; and 0.971—F1-score. Although case 5 is shown to be the lowest performer with 0.722—accuracy and recall; 0.734—precision; and 0.713—F1-score, the performance has tremendously improved. The k-NN with SMOTE has a minimum accuracy of 72% and a maximum of 97%. The k-NN with T-link presents case 6 with the highest performance of 0.9—accuracy and recall; 0.976—precision; and 0.9—F1-score. Cases 4 and 5 present very slight improvements, with case 5 being the lowest performer. The k-NN with T-link has a minimum of 45% and a maximum of 90% accuracy. The k-NN with combined SMOTE/T-link presents extreme improvements for all the cases. Cases 2 and 6 presented the highest performance of 0.972—accuracy and recall; 0.976—precision; and 0.972—F1-score. The k-NN with combined SMOTE/T-link has a minimum accuracy of 71% and a maximum of 97%.

### 4.3. Discussions

In this study, T-link was performed in a data-handling pipeline to address imbalanced data in both simulation and experiments. For the simulation testing, the NBC performance after applying T-link presents improved results compared to original imbalanced data and NBC with SMOTE. The combination of SMOTE and T-link provided improved results in some cases, and in other cases, the performances present improvement compared to SMOTE results and underperformance compared to T-link. For experimental testing, the NBC with SMOTE presented improved and higher performance results compared to T-link and also outperforms the combination of SMOTE/T-link. The NBC with resampling methods for simulated and experimental data has a minimum of 40% and 50% and a maximum of 78% and 75%, respectively.

In the SVM with SMOTE application for simulation, the performance measurements present average improvement for other cases, and for some cases, they perform similar to the original imbalanced data. Even with the combination of SMOTE and T-link sampling methods, in this study, there are cases where the performance is similar to the original. On experimental data, the SVM with SMOTE showed improvements in each case, which outperforms the SVM with T-link which presented case 6 to be performing below the SVM without T-link. The SVM with the combination of SMOTE/T-link outperforms individual methods. The SVM with resampling methods for simulated and experimental data has a minimum of 36% and 39% and a maximum of 78% and 65%, respectively.

In the k-NN with original imbalanced data, the performance measures range from 33.3% to 50%. After the application of SMOTE and T-link, the performance increased for various cases and test data. With the combined SMOTE and T-link, the performances present improvement for each case. On experimental, the k-NN with SMOTE presented the best performance results compared to the k-NN with T-link. The k-NN with the combined methods outperforms individual methods. The k-NN with resampling methods for simulated and experimental data has a minimum of 57% and 71% and a maximum of 84% and 97%, respectively.

The application of NBC, SVM, and k-NN classification algorithms to an imbalanced distribution affects the performance of the classification. The introduction of SMOTE and T-link resampling methods improved the performance of these classifiers. The T-link method in this investigation presented superior performance compared to SMOTE for simulated data. With experimental data, the SMOTE was superior compared to T-link. The SVM classification presented a poor performance compared to NBC and k-NN for both the simulated and experimental data. The integration of SMOTE and T-link outperforms the individual sampling methods. The T-link method performs as post-processing cleaning data by eliminating the majority and minority classes to provide well-defined borderlines. Then, SMOTE is performed with clear regions which provide improved performance for each classification method.

## 5. Conclusions

Data-driven approaches have become attractive in condition monitoring on electrical machines, as they offer the potential benefits of flexibility, scalability, and relatively quicker and cheaper development. Within the data-driven approaches, the supervised learning methods are accuracy-driven and adjust the overall accuracy with minimum errors and ignoring the distribution of each class. The classifier’s accuracy effectiveness depends on even distribution for each class. The misclassification of abnormalities can be costly. The challenge of data imbalance remains an issue for supervised learning approaches—the classifier becomes biased and favors the majority class. There is typically a lack of fault data for different fault classes in practice, which hinders the proliferation of supervised learning-based condition monitoring systems for electrical rotating machines, such as the WRIG. This paper address this challenge and presents a comparative analysis of fault data imbalance approaches, based on different classifiers. Several key metrics are used to evaluate the approaches when applied on different levels and types of WRIG-fault-data imbalance. The NBC, SVM, and k-NN classifiers are compared when used in conjunction with the SMOTE, T-link, and a combination of SMOTE and T-link methods on combined features for fault classification. The evaluation metrics were presented, and the analysis of the above-mentioned classifiers indicates improved performances after the application of SMOTE, T-link, and a combination of the resampling methods. Based on the WRIG’s simulated and experimental imbalanced data under investigation, the accuracies of each of the tested classifiers with the resampling methods are as follows:

1. The NBC with resampling methods for simulated and experimental data has a minimum accuracy of 40% and 50% and a maximum of 78% and 75%, respectively.
2. The SVM with resampling methods for simulated and experimental data has a minimum accuracy of 36% and 39% and a maximum of 78% and 65%, respectively.
3. The k-NN with resampling methods for simulated and experimental data has a minimum accuracy of 57% and 71% and a maximum of 84% and 97%, respectively.

Although the k-NN classifier, when tested with the presented resampling methods, shows the best overall performance for both simulated and experimental data cases, it is found that the combination of the SMOTE and T-link resampling methods does yield improved performance across all classifiers.

## Figures and Tables

**Table 2:** Summary of tested cases indicated number of examples per class (or condition on WRIG).

Description	Class	Case 1	Case 2	Case 3	Case 4	Case 5	Case 6
Healthy	0	30	30	30	30	30	30
Brush	1	30	18	12	9	6	24
Inter-turn short stator—3	2	30	15	9	7	6	18
Inter-turn short stator—6	3	30	9	6	6	6	9
Inter-turn short rotor—3	4	30	9	8	7	6	18
Inter-turn short rotor—6	5	30	7	6	6	6	6

## Data Availability

Not Applicable.
